# Innovative Application of a Multifunctional Sucrose–Gelatin Hydrogel Matrix in Desorption Electrospray Ionization-Mass Spectrometry Imaging

**DOI:** 10.1021/acs.analchem.5c04063

**Published:** 2025-11-07

**Authors:** Marcello Ziaco, Giovanni Andrea Vitale, Giusi Barra, Brenda Marfella, Mario dell’Isola, Federica Albiani, Angela Grazioso, Giuliana Giamundo, Genoveffa Nuzzo, Emiliano Manzo, Carmela Gallo, Daniela Castiglia, Lucia Verrillo, Maria Giuseppina Miano, Luigia Cristino, Ivan Conte, Giuliana d’Ippolito, Angelo Fontana

**Affiliations:** a 201790 Institute of Biomolecular Chemistry (ICB), National Research Council (CNR), Via Campi Flegrei 34, Pozzuoli 80078, Italy; b Department of Biology, 111205 University of Napoli “Federico II”, Via Cupa Nuova Cinthia 21, Napoli 80126, Italy; c Institute of Genetics and Biophysics (IGB), National Research Council of Italy (CNR), Via Pietro Castellino 111, Napoli 80131, Italy

## Abstract

Desorption electrospray ionization-mass spectrometry imaging (DESI-MSI) is among the most powerful techniques for visualizing the spatial distribution of small organic molecules, particularly lipids, on tissue surfaces. Conventional DESI-MSI analysis typically involves sectioning fresh-frozen tissues or, less commonly, embedding samples in matrices specifically formulated to preserve the tissue integrity for multifunctional analyses. In this study, we present an optimized sucrose-gelatin hydrogel matrix compatible with DESI-MSI, using mouse brain tissue as a model system. The method involves low-temperature embedding of frozen specimens into the hydrogel matrix, followed by snap-freezing at −160 °C. This matrix formulation ensures minimal background interference and prevents metabolite delocalization, thereby preserving the native molecular composition of the tissue. Notably, sucrose-derived adduct ions restricted to the embedding medium serve as stable internal reference signals in both positive and negative ionization modes. These signals enable continuous lock-mass correction throughout acquisition, offering a new solution to the unresolved challenge for accurate mass-based measurements in DESI-MSI without an infusion of exogenous calibration standards. Complementary DESI-MS/MS analyses further facilitate confident lipid identification and resolve structural ambiguities. Moreover, the sucrose–gelatin embedding medium provides excellent preservation of tissue morphology and antigenicity, supporting subsequent histological and immunohistochemical analyses. Overall, this sucrose-based hydrogel embedding protocol offers a robust, reproducible, and multimodal platform for molecular tissue imaging by DESI-MSI, especially in delicate biological specimens with broad translational potential across preclinical and clinical research domains.

## Graphical abstract

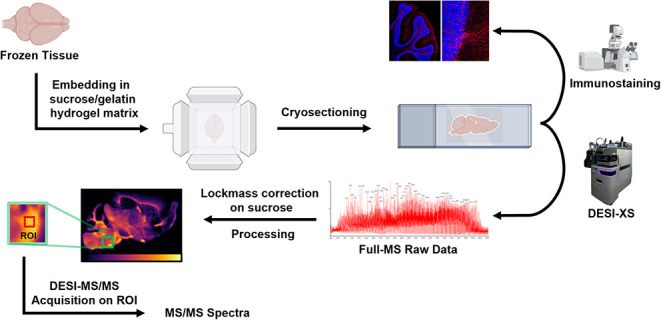

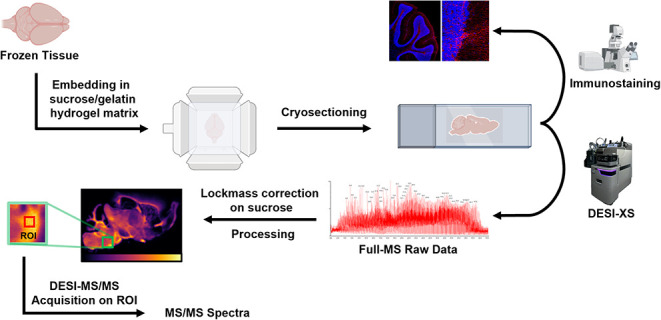

## Introduction

Mass spectrometry imaging (MSI) is a powerful analytical technique for spatially resolving the molecular compositions of biological tissues. By integrating the molecular specificity of mass spectrometry with spatial localization, MSI enables the simultaneous detection and mapping of hundreds of analytes, including proteins, peptides, lipids, and metabolites within intact tissue sections. This approach has found wide-ranging applications across clinical research, biology, pharmacology, food safety, environmental sciences, and forensic analysis.^1–3^

Desorption electrospray ionization (DESI)-MSI is a technique particularly well-suited for the analysis of small organic molecules (50–3000 Da), such as metabolites, lipids, and pharmaceuticals.^4–12^ In contrast to matrix-assisted laser desorption/ionization (MALDI-MS) and secondary ion mass spectrometry (SIMS), DESI-MSI operates under ambient conditions, atmospheric pressure, and room temperature, eliminating the need for extensive sample preparation and preserving the native chemical environment of the tissue. DESI-MSI is typically performed on tissue sections 10–25 μm thick. Charged solvent microdroplets, assisted by a nebulizing gas, are electrosprayed across the tissue surface, desorbing analytes, which are subsequently ionized and transferred via a heated inlet into the mass spectrometer for analysis. By rastering the spray in two dimensions and acquiring pixel-wise mass spectra, the technique allows to reconstruct a high-resolution molecular image. Spatial resolution, typically ranging from 50 to 200 μm as lateral pixel size, depends on the spray spot size, mass spectrometer scan rate, and geometric alignment of the spray and transfer inlet.^13–16^

A critical challenge in DESI-MSI is the maintenance of accurate mass-to-charge (*m*/*z*) measurements, which are essential for reliable molecular annotation. High-resolution mass spectrometers, including time-of-flight (ToF) and Orbitrap analyzers, are sensitive to gradual shifts in mass calibration caused by thermal drift, pressure fluctuations, and ion optical instabilities. Such deviations can compromise the correct assignment of analytes, particularly in complex biological matrices. One widely adopted solution is lock-mass correction, which compensates for mass drift during acquisition.^17,18^ The approach involves continuously monitoring a known reference ion with a precise *m*/*z* value. During data processing, the instrument software uses this reference ion as an internal calibration standard to adjust all acquired m/z values.^19–21^ This enables subppm mass accuracy across entire imaging data sets, thereby enhancing both confidence in molecular identifications and overall data quality. In DESI-MSI workflows, external lock-mass standards (e.g., leucine enkephalin at *m*/*z* 556.2771 in positive mode and *m*/*z* 554.2615 in negative mode) are often coinfused with the spray solvent. While effective, this approach is not without limitations since exogenous compounds can cause ion suppression, compete with endogenous metabolites for ionization, and introduce chemical noise or contamination that affects reproducibility.^21^

To achieve a comprehensive understanding of tissue biochemistry, DESI-MSI data must often be integrated with other imaging modalities, including histological staining and immunohistochemistry.^22,23^ This necessitates sample preparation protocols that preserve tissue morphology, spatial orientation, and molecular integrity across multiple analytical platforms. Small and fragile tissues, in particular, demand optimized embedding strategies that support both cryosection and downstream analysis. Common embedding media, such as formalin-fixed paraffin-embedding (FFPE), optimal cutting temperature compound (OCT), and carboxymethylcellulose (CMC), are primarily designed for histological processing and are often incompatible with MSI due to matrix-related ion suppression and spectral interference. Hydrogel-based matrices, including hydroxypropyl methylcellulose (HPMC), polyvinylpyrrolidone (PVP), and gelatin-based formulations, have recently emerged as more MSI-compatible alternatives.^24–28^

Sucrose–gelatin matrices have shown particular promise in preserving soft and delicate biological specimens, especially for histological and microscopic applications.^29–31^ Gelatin, a collagen-derived protein, imparts structural integrity and mechanical support during sectioning, while sucrose enhances the matrix stability by reducing ice-crystal formation during freezing. Additionally, sucrose improves epitope preservation, thereby facilitating consistent immunostaining. Together, sucrose and gelatin form a robust, cryosectionable embedding medium that stabilizes the tissue morphology without compromising molecular fidelity.

In this study, we evaluated the compatibility of a sucrose–gelatin hydrogel matrix for DESI-MSI of biological tissues using mouse brain as a representative soft tissue model. The embedding protocol involved the optimized incorporation of sucrose into gelatin, low-temperature tissue embedding, and snap-freezing at −160 °C. DESI-MSI was employed to assess the matrix background interference, analyte delocalization, and preservation of native molecular distributions. Brain lipid profiling served as a test case to validate the capacity of the protocol to maintain both molecular and morphological integrity. Key metrics included preservation of tissue architecture, antigen reactivity, and spatial fidelity of endogenous lipids during DESI-MS imaging.

The entire procedure has been validated by analysis of representative lipids of a soft tissue such as mouse brain. The key objectives were to determine whether the cryosections of embedded matrix tissues resulted in preserving tissue integrity and antigen reactivity, maintaining the native molecular distribution of lipids, and minimizing background interference during DESI-MS imaging.

## Experimental Methods

### Materials

Methanol (LC–MS grade), acetone (HPLC plus grade), water (LC–MS grade) sucrose, PBS, gelatin type A, Triton X-100, DAPI (4′,6-diamidino-2-phenylindole), Aquatex mounting medium, and paraffin mold (16 cm^3^) were purchased from Merk Life Science S.r.l. (Milan, Italy). Paraformaldehyde cat. no. 3162802, used for fixation, was purchased by SERVA Electrophoresis GmbH (Heidelberg, Germany) while MajorMix Part No. 186008113-2 used for mass spectrometer calibration was supplied by Waters (Manchester, UK).

### Animals and Ethical Statements

The study was carried out according to the ARRIVE Guidelines to improve the reporting of bioscience research using laboratory animals. All efforts were made to minimize animal suffering and reduce the number of animals used. All experiments in the mice were conducted in accordance with the principles of the European Union animal welfare guidelines [European Communities Council Directive of September 22, 2010 (2010/63/EU)] and the Italian Decree no. 26/2014, under the accreditation no. 307/2018-PR E58D.8 and authorization no. 783/2020, by using C57BL/6 mice purchased from the Charles River Laboratories Int.© (Wilmington, Massachusetts, USA). The mice received chow and tap water *ad libitum* and were housed under controlled illumination (12 h light/dark cycle; light on at 6:00 am, ZT0) and environmental conditions (ambient temperature 20–22 °C, humidity ∼50%) before sacrifice at age 8–10 weeks using CO_2_ followed by cervical dislocation at ZT4–ZT6.

### Matrix Preparation and Cryosectioning

100 mL of PBS was heated to 55 °C on a magnetic stirrer. Sucrose (15 g, 15% w/v) was added to PBS under vigorous stirring. Then, 7.5 g of granular gelatin (7.5% w/v) was added, and the mixture was vigorously stirred for at least 30 min to obtain a homogeneous solution. After preparation, the solution was cooled and maintained at temperature >37 °C to ensure fluid consistency until using for tissue embedding. The sucrose–gelatin medium was transferred in a mold that was snap frozen in a box containing isopentane and chilled at −160 °C in liquid nitrogen. The frozen blocks were stored at −80 °C until sectioning. Sections (thickness 20 μm) were obtained using a CryoStar NX50 Cryostat (Thermo Fisher) or Leica CM3050S (Leica Microsystems, Wetzlar, Germany) collected on slides (Superfrost Plus; Fisher Scientific, Pittsburgh, PA). Slides were stored in an −80 °C freezer until DESI analysis.

### Brain Sampling, Embedding, and Cryosectioning

Whole murine brains were quickly removed from the skull and immediately frozen in a dish plate and put in a box over dry ice. Frozen tissues were stored at −80 °C until incorporation into the gel-based matrix. Each frozen brain wasplaced in molds containing the sucrose–gelatin medium at 37 °C using tweezers for the desired orientation. The entire mold containing the brain was snap frozen in a box containing isopentane and chilled at −160 °C in liquid nitrogen. The frozen blocks were transferred in dry ice for ensuring isopentane evaporation and then stored in a −80 °C freezer until sectioning. All of the operations were performed under a fume hood. Sagittal or coronal brain cryosections (thickness 20 μm) were obtained using a CryoStar NX50 Cryostat (Thermo Fisher) or Leica CM3050S (Leica Microsystems, Wetzlar, Germany) collected in alternate series on slides (Superfrost Plus; Fisher Scientific, Pittsburgh, PA) stored at −80 °C until analysis. Coronal sections at distance from Bregma ranging from −1.2 to −2.0 mm and sagittal sections at −0.02 to −0.20 mm from the middle line of the brain were selected for our study.

### DESI-MSI Data Acquisition

MSI experiments were performed on a time-of-flight (ToF) mass spectrometer (SYNAPT-XS, Waters, Manchester, UK) equipped with a DESI-XS (Waters, Manchester, UK). Prior to analysis, the slides were retrieved from the −80 °C freezer and placed into a vacuum desiccator for 10 min, to reach room temperature and to eliminate humidity

The parameters of the DESI sprayer were optimized for the best signal intensity on marker pens: solvent, methanol/water 98:2 (v/v); flow rate, 0.5 μL/min; DESI modality, positive and negative mode; capillary voltage, 0.40 kV; sampling cone voltage, 40 V; spray incidence angle, 75°; sprayer-to-inlet distance, 2 mm; and sprayer-to-sample distance, 2 mm; and nebulizing gas (N_2_) pressure, 1.15 bar. The mass spectrometer was operated in “resolution mode”. The source temperature was set to 150 °C and the Heated Transfer Line to 300 °C. The ToF-MS was operated over the *m*/*z* range of 100–1200, with a single scan time of 0.5 s. Specific parameters for matrix sections: scan time, 0.653 s/pixel; pixel size, 100 μm^2^; specific parameters for brain embedding tissue sections: scan time, 0.153 s/pixel, pixel size lateral of 25 μm. All imaging data were acquired using HDI Imaging Version 1.8 in combination with Masslynx Version 4.2 (Waters, Manchester, UK). As a reference mass to perform the lock-mass correction, we used the deprotonated chlorinated adduct ion of sucrose ([M+Cl]^−^, *m*/*z* 377.0856) in negative mode and the sucrose adduct ([M + Na]^+^, *m*/*z* 365.1054) in positive mode, composing the matrix used to embed the tissue for the section. For processing in the HDI software, in the lock-mass column, lock mass was enabled using *m*/*z* 377.0856 (negative ion) and *m*/*z* 365.1054 (positive ion), lock-mass tolerance set at 0.25 aum, and min signal intensity at 500 counts.

### DESI-MS/MS Data Acquisition and Analysis

After DESI-full-MS acquisition of the sections, selected ions were analyzed by DESI-MS/MS on the same slides. MS/MS spectra were recorded in regions of interest (ROIs) where the specific molecules are particularly abundant in full-MS DESI analysis. The MS file on Masslynx was edited to set the collision voltage energy ramp (10–70 V) on the Trap CE control panel. Over an area of 0.30 mm^2^, the scan time was 0.153 s/pixel, with a pixel size lateral of 25 μm, and the nebulizing gas (N_2_) pressure was at 1.15 bar. The ToF-MS was operated over the *m*/*z* range of 50–1200.

### Tissue Staining and Microscopy

The brain sections previously acquired by DESI-MSI or adjacent to those imaged by DESI were quickly fixed by immersion in 4% (w/v) paraformaldehyde/0.1 M phosphate buffer (PB), pH 7.4, to be processed for immunofluorescence. To this purpose, the sections were incubated 1 h at room temperature (RT) in the PB solution containing 0.3% Triton X-100 and 5% normal donkey serum (Abcam, cat. no. ab7475) before being incubated overnight at 4 °C in a PB solution containing mouse anti-GFAP (Invitrogen, cat. no. MA5-12023—Astro6; working dilution = 1:500) primary antibody. Immunofluorescence was revealed by a 2 h incubation RT of sections with PB solution containing Alexa-546 secondary donkey anti-IgGs (Invitrogen, ThermoFisher Scientific, France; working dilution = 1:300). The sections were then washed in PB before being stained for nuclear labeling with DAPI, 3 min at RT in a humidified chamber. Finally, the sections were coverslipped with an Aquatex mounting medium. Control of specificity for immunoreactivities was performed by omission of primary or secondary antibodies or by preabsorption of the primary antibody with selective blocking peptide.

The immunostained sections were observed with a Leica TCS SP5 confocal microscope (Leica^©^, Germany) equipped with an x-y-z motorized stage. Digital serial Z-stacks of images were acquired throughout the area of interest (*n* ≤ 10 planes with an increment varying 0.5–1 μm). Images were processed using the Leica MetaMorph imaging deconvolution software (Leica^©^, Germany). Micrographs were saved in TIFF format and adjusted for light and contrast before being assembled in the figure by using Adobe Photoshop 6.01 (Adobe Systems, San Jose, CA).

To assess tissue morphology, GPAF, and/or DAPI nuclei labeling, the brain sections were imaged in brightfield or phase-contrast or confocal microscopy. Brighfield acquisition of Nissl staining or phase-contrast imaging were performed by a Leica DMI6000 microscope (Leica^©^, Germany) equipped with an x-y-z motorized stage and Leica K5 digital camera (Leica^©^ Germany). High-resolution mosaic images were generated by capturing multiple 5× objective fields from the same section and stitching them using standard algorithms.^59^ Micrographs were saved in a TIFF format and adjusted for brightness and contrast. Brain regions of interest were identified by referencing to The Mouse Brain atlas.^58^

## Results and Discussion

### Chemical Background of Sucrose–Gelatin Embedding Medium in DESI-MSI

Preserving tissue morphology and molecular integrity is essential in mass spectrometry imaging (MSI), particularly for soft, small, and fragile samples. A suitable embedding medium must enable effective cryosection without compromising the native molecular composition.

We evaluated the compatibility of a sucrose–gelatin-embedded medium for DESI-MSI. A homogeneous solution of 15% (w/v) sucrose and 7.5% (w/v) gelatin was prepared in the phosphate-buffered saline (PBS) and snap frozen at −160 °C in a molding box. The blocks were sectioned at 20 μm thickness and analyzed by DESI-MSI with a resolution of 100 μm as lateral pixel size

Full-MS experiments (50–2000 Da) at different section depths revealed a clean, homogeneous, and reproducible ion profile primarily composed of sucrose-related ions. In negative ion mode, the main sucrose adducts included [M–H]^−^ (*m*/*z* 341.1095), [M + Cl]^−^ (*m*/*z* 377.0883), and [2M–H]^−^ (*m*/*z* 683.2227), with the chlorinated adduct being the most abundant.^32^ In positive ion mode, the predominant ion was [M + Na]^+^ (*m*/*z* 365.1057), followed by [2M + Na]^+^ (*m*/*z* 707.2209) and [3M + Na]^+^ (*m*/*z* 1049.3373) (Figure 1). Notably, no gelatin-derived ions were detected in the selected *m*/*z* range. Data were validated on four independent preparations confirming the reproducibility of the ionization profile (Supporting Information, Figure S1). Sucrose-based fingerprinting of the embedding medium suggested a highly reliable use of these ions as reference in the lock mass correction.

**Figure 1:**
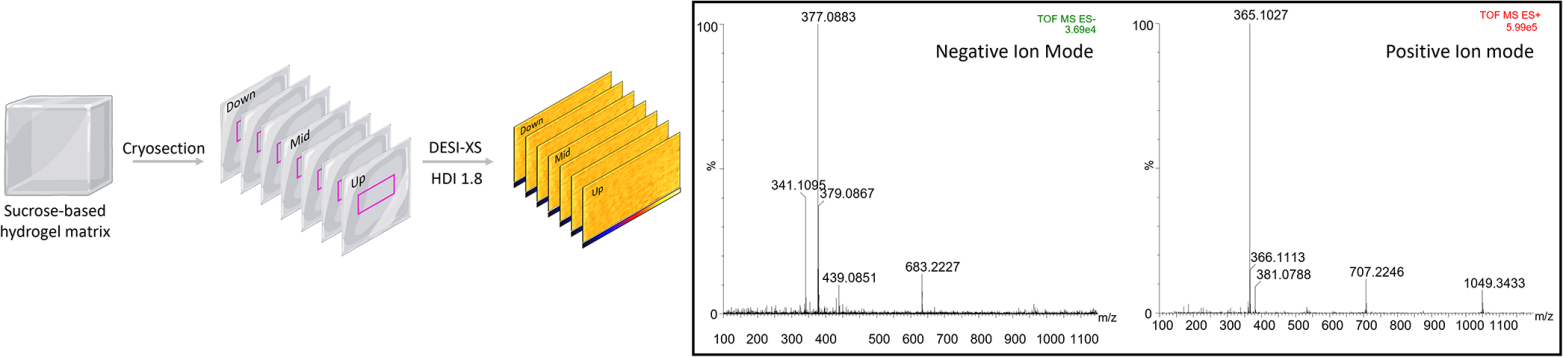
Schematic workflow of DESI-MSI analysis on sections of sucrose–gelatin matrix blocks, showing full-MS spectra in negative and positive ion modes.

### Embedding Tissue in DESI-MSI

Once established the stable and homogeneous ionization of the sucrose–gelatin hydrogel matrix in DESI-MSI, we evaluated its performance in a biologically relevant context by embedding and analyzing mouse brain as a case study of soft and fragile tissue. Frozen mouse brains were carefully placed with appropriate orientation into molds containing the sucrose–gelatin medium at 37 °C. The blocks were snap frozen in isopentane at −160 °C and chilled in liquid nitrogen. The frozen blocks were cryosectioned in coronal or sagittal slices (20 μm thickness) for subsequent DESI-MSI analysis.

Full DESI-MS spectra were acquired in both ion modes over the 100–1200 Da range at 25 μm as lateral pixel dimension. Sucrose adducts were strictly confined outside tissue regions with no matrix-related ions infiltrating the tissue interior. This is evident comparing the full spectra of two closely aligned ROIs, one outside the tissue and another one inside the tissue (Figure 2). Accurate analysis along the edge of the tissues revealed no significant metabolite diffusion and delocalization into the embedding medium, supporting the high containment effect operated by the matrix. Data suggest that the sucrose-based hydrogel matrix remains confined to the embedding area without infiltrating the tissue and maintaining the native molecular environment.

**Figure 2:**
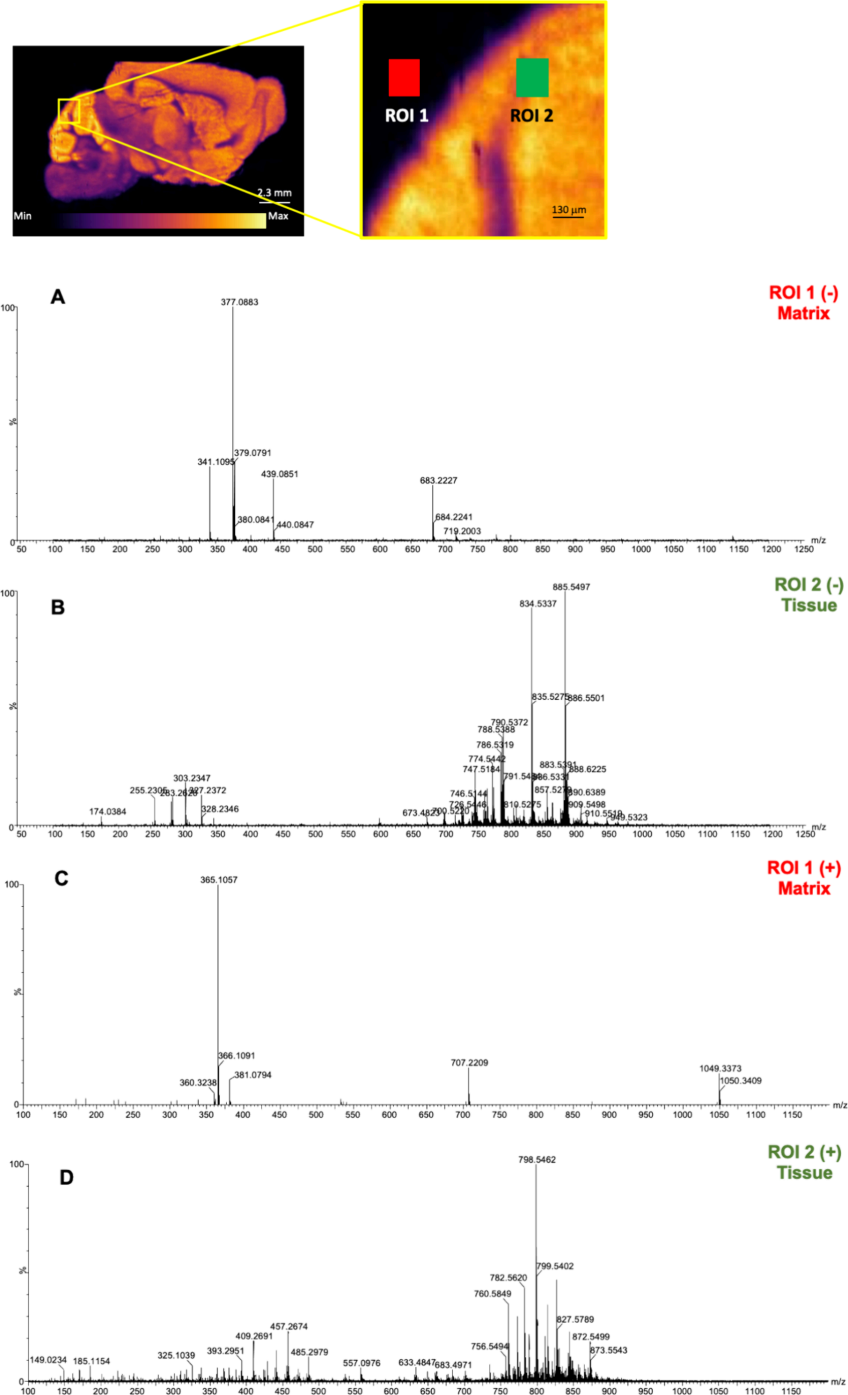
Full-MS spectra obtained from two aligned ROIs in the cerebellum region of a sagittal section of a mouse brain embedded in the sucrose–gelatin matrix acquired by DESI-MSI. ROI 1 (red square) is located outside the tissue in the embedding matrix, and ROI 2 (green square) is located inside the tissue. (A) ROI 1, negative ion mode; (B) ROI 2, negative ion mode; (C) ROI 1, positive ion mode; (D) ROI 2, positive ion mode.

### Leveraging the Matrix for Lock-Mass Correction

Mass accuracy is critical for reliable molecular annotation in MSI, particularly during long imaging acquisitions. Lock mass correction enables calibration of mass measurements using known ion references.^21^ The exclusive detection of sucrose adducts ions in the embedding medium suggested the opportunity to apply lock mass correction using sucrose as a reference compound in both positive and negative ion modes, without the need for introducing exogenous standards affecting the native environment.

In detail, after definition of specific imaging patterns including portions of embedding medium, DESI-MSI acquisitions proceeded by collecting data horizontally row by row as defined by the selected pixel lateral size. Since the embedding medium surrounds the tissue like a frame, every acquisition row begins and/or ends in regions containing the sucrose–gelatin hydrogel. As a result, sucrose-derived ions are consistently detected in each line scan, providing reliable internal references for row-wise lock-mass correction and ensuring stable mass accuracy throughout the experiment.

We validated this approach using major sucrose adducts as lock mass references, being [M + Na]^+^ ion (*m*/*z* 365.1054) and [M+Cl]^−^ (*m*/*z* 377.0856) for positive and negative ion modes, respectively. Higher mass calibration was also achieved by using sodium adducts [2M + Na]^+^ (*m*/*z* 707.2209) and [3M + Na]^+^ (*m*/*z* 1049.3373) in positive mode and deprotonated adduct [2M–H]^−^ (*m*/*z* 683.2227) in negative ion mode, ensuring precision across wider *m*/*z* ranges.

Leucine enkephalin infusion during DESI-MSI acquisitions is the most common approach for using the peptide as an external lock-mass standard to correct for mass inaccuracies. Comparison of TIC spectra obtained on fresh-frozen brain sections acquired using leucine enkephalin infusion and sucrose–gelatin-embedded sections revealed higher signal intensities and low chemical background in the latter case (Supporting Information, Figures S2–S3). The combined spectrum of a single acquisition row, extracted at the same retention time from corresponding anatomical areas in both conditions, further demonstrated an improved signal-to-noise profile in embedded tissues, largely due to the absence of dominant leucine enkephalin signals. In line with this, the comparison of extracted ion chromatograms (XICs) of some representative lipids corroborated the finding, underscoring the overall enhancement in signal quality achieved with the embedding approach (Supporting Information, Figures S4–S5).

### Distribution of Representative Lipid Classes in the Mouse Brain

To assess the ability of the embedding medium to preserve lipid spatial distribution, we analyzed different lipid classes in embedded mouse brain tissues. Representatives of phosphatidylinositols (PI), phosphatidylethanolamines (PE), phosphatidylserines (PS), sulfatides (ST), phosphatidylcholines (PC) and cholesterol were mapped on coronal and sagittal sections, in positive and negative ion modes, with a resolution of 25 μm^2^ across a mass range of 100–1200 Da. The selected lipid classes are recognized functional or structural components of the central nervous system (CNS).

In addition, their well-documented specific localization within gray and white matter underlines their role in neuronal function in specific areas of the brain.^33,34^

PIs are critical signaling molecules and regulate intracellular pathways, such as vesicular trafficking, cytoskeletal remodeling, and synaptic signaling. In negative ion mode, PI (18:0/20:4) (*m*/*z* 885.5499), PI (18:1/20:4) (*m*/*z* 883.5354), and PI (38:3) (*m*/*z* 887.5684) were localized to gray matter regions, including the amygdala, hippocampus, thalamus, and cortex (Figure 3a). PI (39:0) (*m*/*z* 907.6303) was more concentrated in white matter structures, such as corpus callosum and fiber tracts. PEs are structural lipids that maintain the curvature and fluidity of cell membranes and are involved in energy metabolism and neurotransmitter release. PE (18:0/18:1) (*m*/*z* 744.5559) showed widespread distribution in the thalamus and white matter, while PE (18:0/22:6) (*m*/*z* 774.5475) localized more selectively to the cerebellum and visual cortex (Figure 3b). PSs are anionic phospholipids primarily located in the inner leaflets of neuronal plasma membranes, where they play a vital role in maintaining membrane integrity and supporting signaling pathways and apoptosis regulation. In agreement with the literature, PS (16:0/18:1) (*m*/*z* 760.5143) and PS (18:0/18:1) (*m*/*z* 788.5465) were found in both gray and white matter regions (Figure 3c).

**Figure 3:**
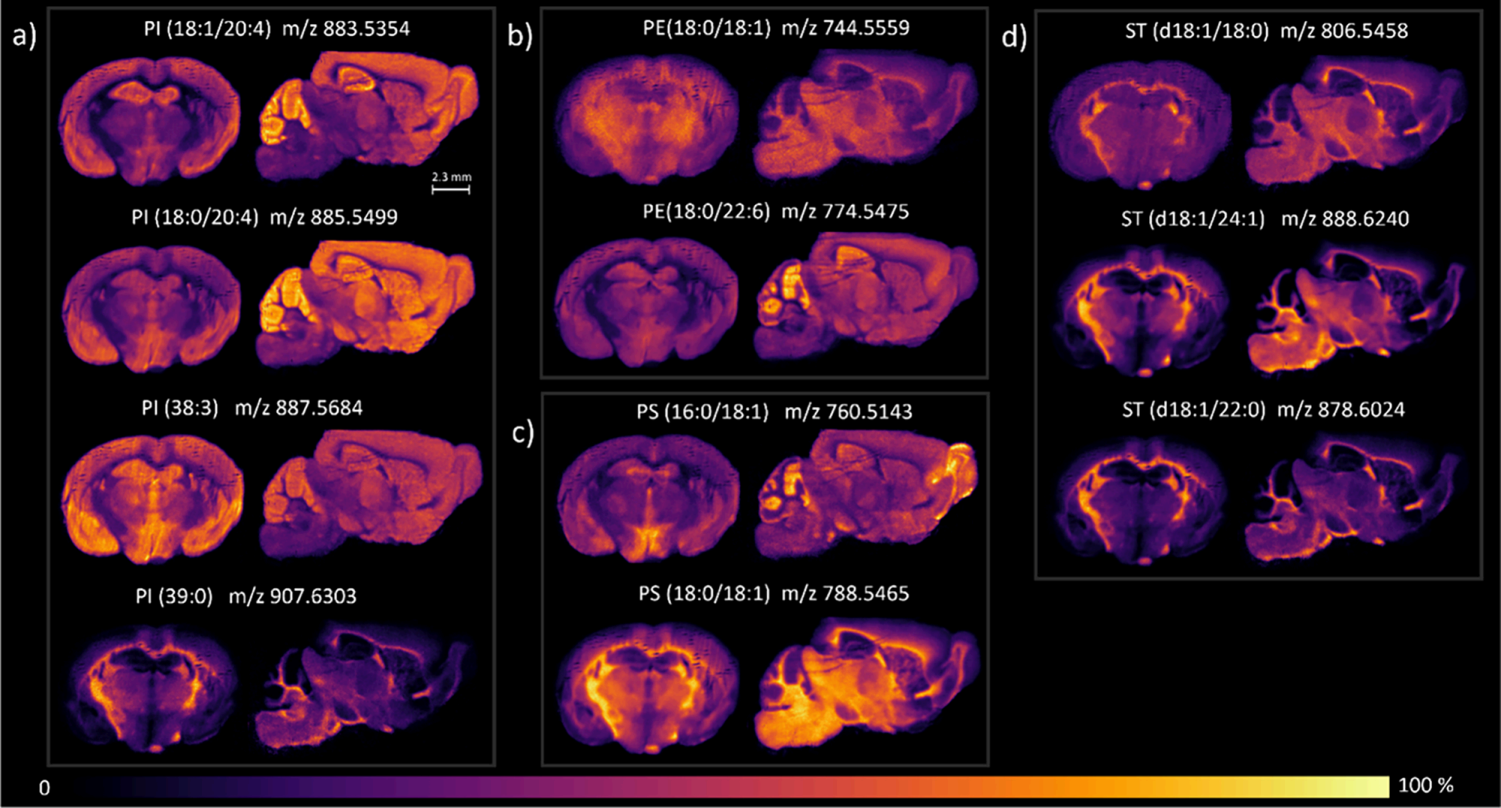
DESI-MS images of some lipid species in coronal (left side) and sagittal sections (right side) of mouse brains embedded in the sucrose–gelatin matrix and acquired in negative ion mode. (a) Phosphatidylinositols (PIs); (b) phosphatidylethanolamines (PEs); (c) phosphatidylserines (PSs); and (d) sulfatides (STs). The lateral pixel size of the DESI-MS images is 25 μm, and all of the ion images were TIC-normalized. All of the ions are reported as deprotonated species ([M–H]^−^). The mass accuracy of the selected ions ranged from 1 to 10 ppm, relative to their theoretical *m*/*z* values. Lock mass correction has been made on the sucrose adduct [M + Cl]^−^ (*m*/*z* 377.0856) in negative ion mode.

STs are a subclass of sphingolipids integral to the structure and function of myelin. According to this role, we observed their specific distribution in the white matter of corpus callosum, caudate-putamen, globus pallidus, olfactory tubercle, and fiber tracts (Figure 3d). As previously reported, their distribution within axonal tracts is not uniform, even in the most highly myelinated areas of the brain.^33–37^ Interestingly, while STs such as ST (d18:1/18:0), ST (d18:1/22:0), and ST (d18:1/24:1) were distributed uniformly, ST (d18:1/24:1) (*m*/*z* 888.6240) was enriched in white matter and motor coordination nuclei, reflecting their role in myelination (Figure 3d).

PCs are some of the most abundant phospholipids in the central nervous system (CNS). PCs supported a range of processes such as synaptic signaling, membrane trafficking, and the dynamic remodeling required for neuronal communication and repair.^38^ Beyond their structural contributions, PCs act as precursors for bioactive lipids, such as lysophosphatidylcholine (LPC) and diacylglycerol (DAG), which are involved in both normal and adaptive responses of neuronal cells during stress or neurodegeneration. In terms of anatomic localization, PCs exhibit a distinctive and complementary pattern distribution in the brain that may reflect their specific roles. PC (34:1) (*m*/*z* 760.5851) and PC (36:1) (*m*/*z* 788.6164) predominated in the white matter of corpus callosum, caudate-putamen, globus pallidus, olfactory tubercle, and fiber tracts, while PC (32:0) (*m*/*z* 772.5296) and PC (16:0/20:4) (*m*/*z* 804.5528) were prominent in gray matter, specifically in brain areas such as cortex, amygdala, thalamus, hypothalamus, and hippocampus (Figure 4a).

**Figure 4:**
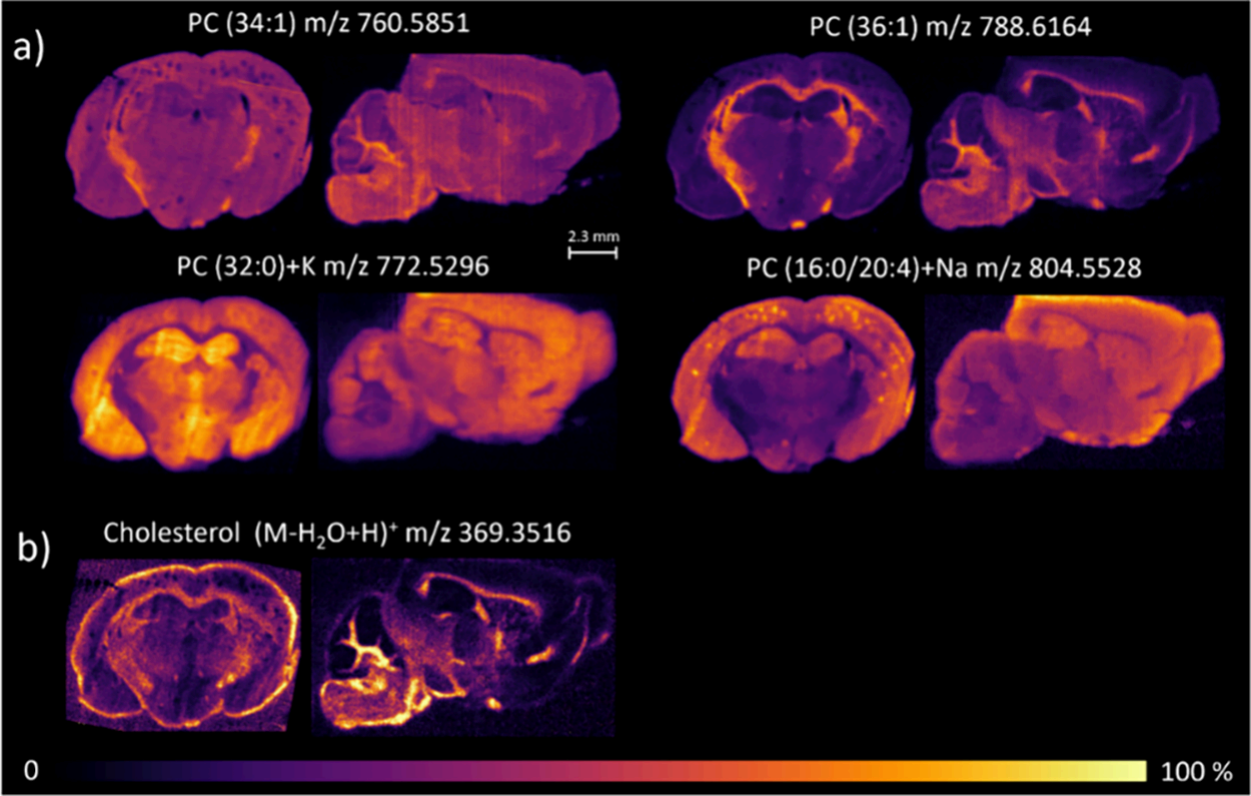
DESI-MS images of some lipid species in coronal (left) and sagittal sections (right) of mouse brains embedded in the sucrose–gelatin matrix and acquired in positive ion mode. (a) Phosphatidylcholine (PC), (b) cholesterol. The lateral pixel size of the DESI-MS images is 25 μm, and all of the ion images were TIC-normalized. PC (34:1) and PC (36:1) were detected as protonated species ([M + H]^+^), whereas PC (32:0) and PC (16:0/20:4) were observed as potassium and sodium adducts, respectively. Cholesterol was detected as a dehydrated protonated ion ([M–H_2_O+H]^+^), and image gain was increased to enhance cholesterol visibility, which also made background noise outside the tissue more apparent. The mass accuracy of the selected ions ranged from 1 to 10 ppm relative to their theoretical *m*/*z* values. Lock mass correction has been made on the sucrose adduct [M + Na]^+^ (*m*/*z* 365.1054) in positive ion mode.

Cholesterol accounts for a significant lipid fraction in white matter, providing the necessary rigidity and insulation for myelin sheaths.^39,40^ Considering its intrinsic ionization properties, cholesterol was detected as the dehydrated protonated ion [M–H_2_O+H]^+^ (*m*/*z* = 369.3516) in MS experiments. We localized this ion by DESI-MSI in ependymal regions, corpus callosum, caudate-putamen, globus pallidus, olfactory tubercle, nuclei pontini, parvicellular and spinal trigeminal nuclei, and fiber tracts (Figure 4b). Despite its notoriously poor ionization,^41–46^ we detected the molecule in our DESI-MSI experiments without derivatization or ionization enhancement strategies.^47–49^ We attribute this outcome to the improved DESI performance as well as to the embedding approach creating a clean background optimal for the detection of minor compounds and poorly ionizable molecules.

### Enhancing Lipid Identification via DESI-MS/MS

Despite the high mass resolution provided by modern MSI platforms, accurate metabolite annotation remains a significant challenge due to the presence of isomeric and isobaric species, which often cannot be resolved by exact mass values. In complex biological tissues such as the brain, where multiple lipids share the same nominal mass but differ in structural configuration, complementation of full-MS data with MS/MS fragmentation experiments can significantly increase the confidence of structural assignment of key metabolites. A clear advantage of the DESI source is its mild ionization mechanism, which enables MS/MS acquisitions on the same tissue section immediately after full-MS imaging.

For this reason, we tested targeted DESI-MS/MS on selected ROIs to confirm the lipid identities of the major compounds (Figure 5). The most abundant PI (18:0/20:4) at *m*/*z* 885.5499 gave consistent fragments at *m*/*z* 241.0132 (inositol headgroup), 152.9967 (phosphoglycerol), 283.2649 (stearic acid), and 303.2331 (arachidonic acid) (Figure 5a). The major ST (d18:1/24:1) at *m*/*z* 888.6240) yielded characteristic ions at *m*/*z* 241.0030 (sulfated galactose), 96.9603 (sulfate), and 540.2888 for the neutral loss of the amide-linked fatty acyl chain (Figure 5b). The most abundant PC (16:0/20:4) (*m*/*z* 804.5528) showed *m*/*z* 184.0736 (phosphocholine) and neutral loss fragments at *m*/*z* 745.4740 and 621.4849 (Figure 5c). Although low in intensity, MS/MS confirmed expected fragments of cholesterol ([M–H_2_O+H]^+^, *m*/*z* 369.3516) (Figure 5d) that were consistent with Global Natural Product Social Molecular Networking (GNPS) database entries (Supporting Information, Figure S6). Unlike conventional imaging approaches that typically rely only on accurate mass, these targeted MS/MS experiments validated full-MS annotations and enabled confident lipid identification based on database searches^50–52^ and chemoinformatic tools.^53–55^

**Figure 5:**
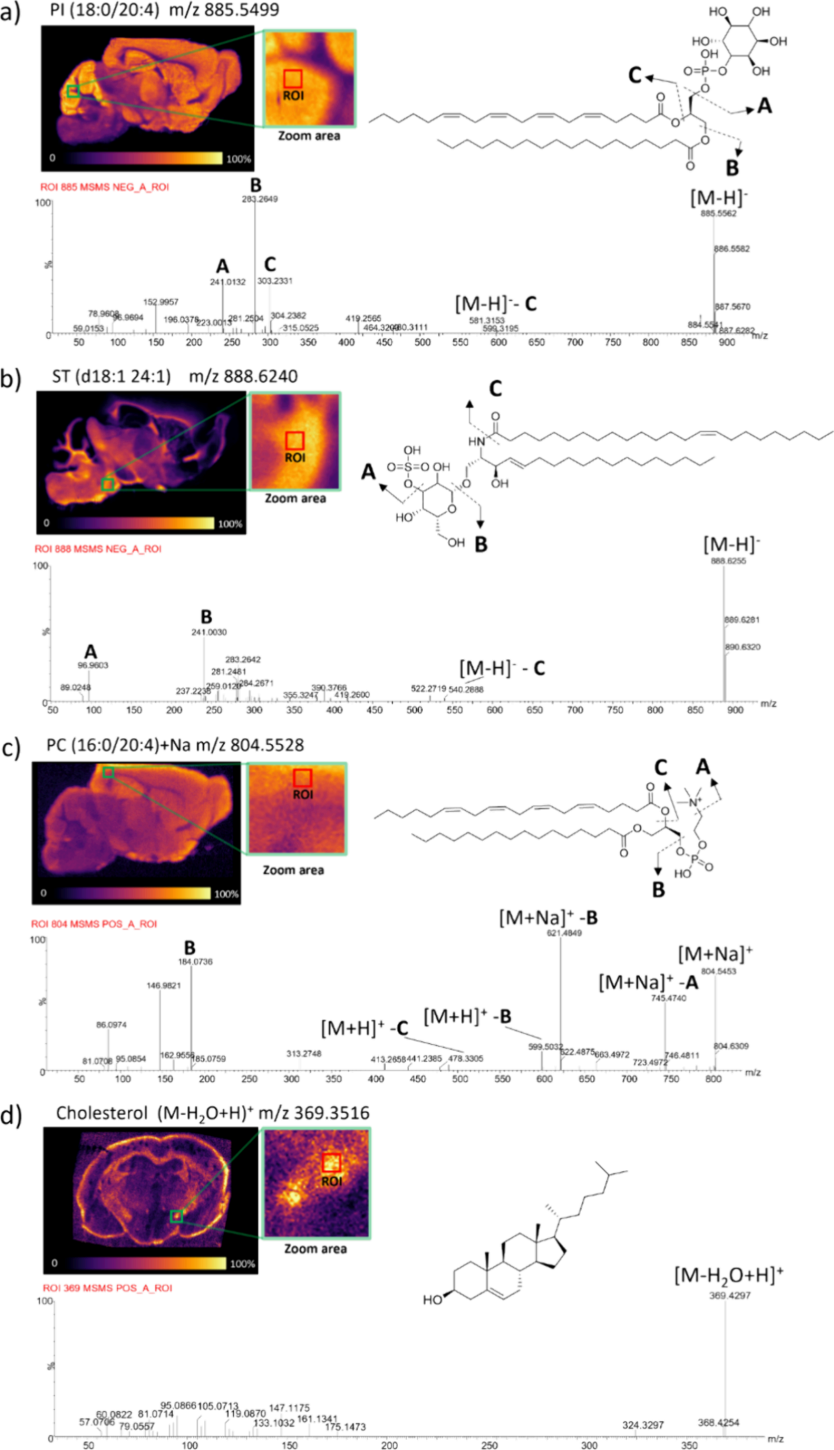
DESI-MS/MS spectra acquired on specific ROIs of coronal and sagittal sections of mouse brains embedded in the sucrose–gelatin matrix. The diagnostic fragments on each spectrum adds confidence to the identification of PI (18:0/20:4) [M–H]^−^ (a), ST (d18:1 24:1) [M–H]^−^ (b), PC (16:0/20:4) [M + Na]^+^ (c), and cholesterol [M–H_2_O+H]^+^ (d).

### Effect of the Embedding Media on Tissue Morphology and Immunoistochemistry

To confirm that our embedding method preserves tissue integrity,^24,25,56,57^ we analyzed morphology and immunoreactivity in sections adjacent to those used for DESI-MSI. Nevertheless, given the gentle desorption mechanism of DESI and the ability of sucrose to preserve epitopes, post-DESI immunofluorescence on the same section should, in principle, be feasible under optimized conditions. Nissl staining showed well-preserved cytoarchitecture in both coronal (Figures 6) and sagittal (Figures 7) orientations, with no ice-crystal damage or fracturing artifacts. Structural integrity extended throughout the sample depth from the outer layers to the central core (Figures 6A and 7A). Immunofluorescence at 5 μm resolution demonstrated excellent compatibility with immunohistochemical analysis. Hippocampal subfields (CA1–3, DG), granular cell layers, and astrocyte populations were clearly visualized via GFAP/DAPI labeling (Figures 6B.1–3 and 7B.1–4).

**Figure 6:**
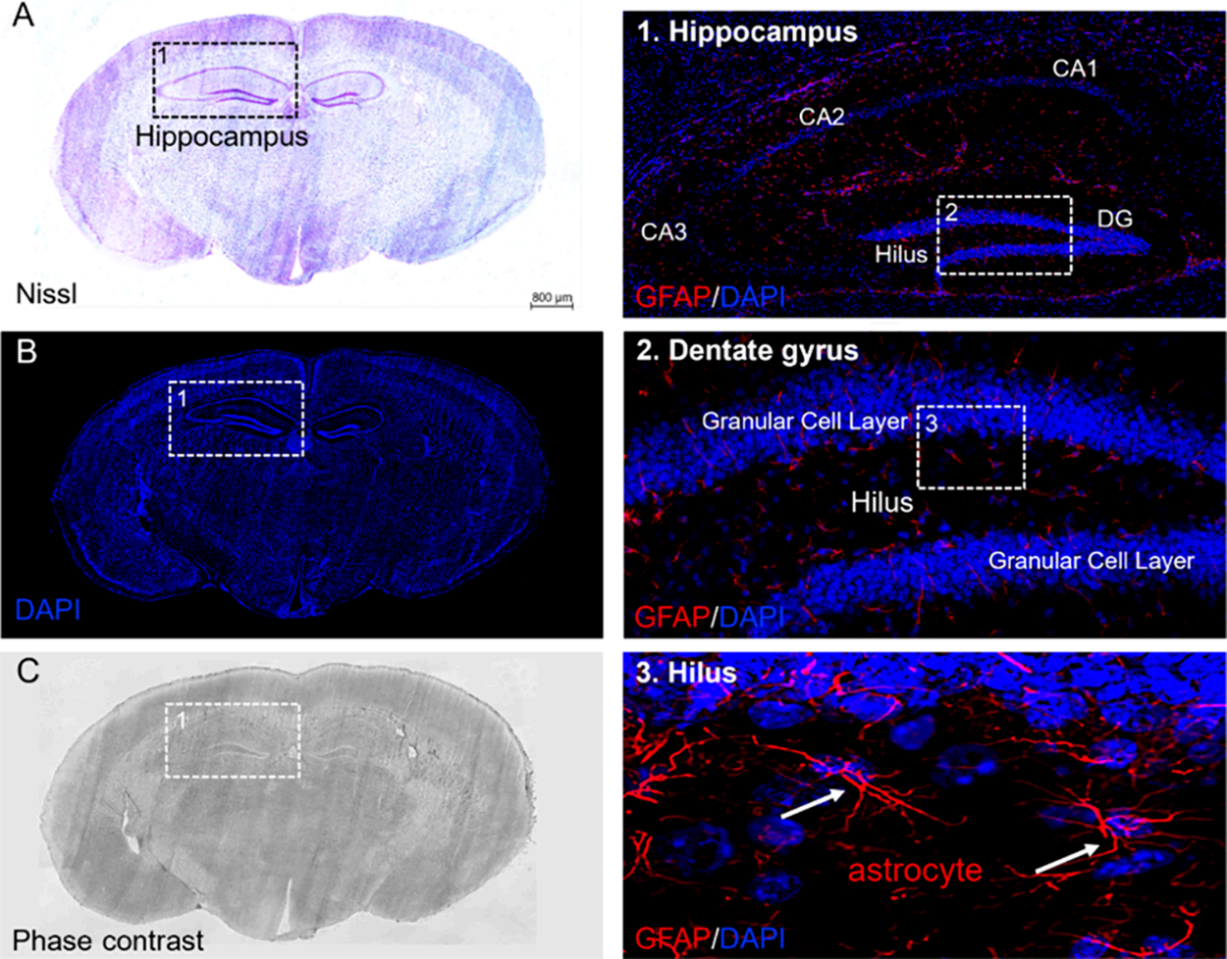
High accuracy of matrix impregnation in preserving brain anatomy, cytoarchitecture, and selective antigenic reactivity at single-cell resolution, as demonstrated by the correspondence between the morphology of the same coronal brain section (Bregma: −1.4 mm) imaged by MSI and the Nissl staining visualized by light microscopy (A). (B) DAPI labeling of nuclei in a brain section 20 μm adjacent from that shown in (A). High-resolution image of the boxed area in (B), showing GFAP/DAPI immunostaining of the dentate gyrus (DG), hilus, and CA1–3 subfields of the hippocampus (1). High magnification of DG in the boxed area (1), illustrating the laminar distribution of GFAP/DAPI immunoreactivity in the granular cell layer and DG (2). Single-cell resolution of GFAP-immunoreactivity astrocytes in the boxed area (2), showing GFAP/DAPI-positive astrocytes (arrows) in the hippocampal hilus (3). (C) Phase-contrast image of the same section from panel (B) showing the white and gray matter compartments of brain tissues. DG: dentate gyrus; CA1–3; Ammons’s horns; scale bar = 800 μm in panels (A–C), 200 μm in panel (1), 100 μm in panel (2), and 10 μm in panel (3).

**Figure 7:**
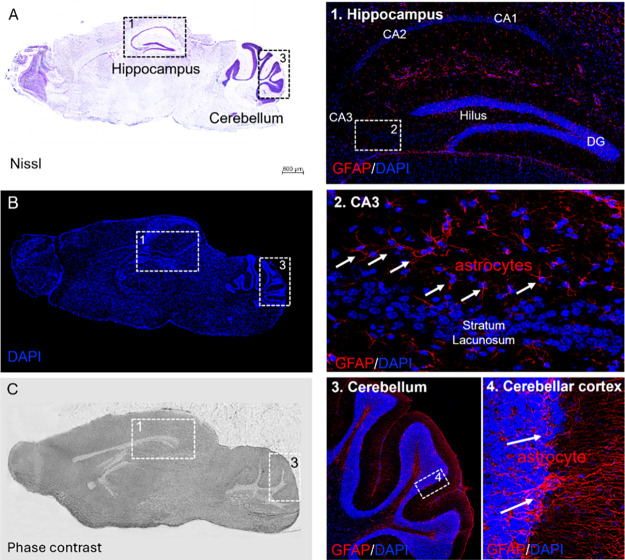
High accuracy of the matrix impregnation in preserving brain anatomy, cytoarchitecture, and selective antigenic reactivity at single-cell resolution as demonstrated by the correspondence between the morphology of the same sagittal brain section (Lateral: −0.04 mm) imaged by MSI and the Nissl staining visualized by light microscopy (A). Brain section at 20 μm adjacent from that shown in (A) imaged by confocal microscopy for DAPI labeling (B) and GFAP/DAPI immunostaining of the dentate gyrus (DG), hilus, and CA1–3 subfields of the hippocampus (1). GFAP/DAPI-reactive details of the laminar distribution of the stratum lacunosum with high single-cell resolution of astrocytes (GFAP-immunoreactive cells; arrows) (2). High magnification of the cerebellum (3) and cerebellar cortex (4). (C) Phase-contrast image of the same section shown in (B), displaying the white and gray matter compartment of the brain tissue. DG: dentate gyrus; CA1–3: Ammons’s horns; scale bar = 800 μm in panels (A–C), 100 μm in panels (1,3) and 20 μm in panels (2,4).

Phase-contrast microscopy further supported preservation of white/gray matter architecture, consistent with canonical brain atlases (Figures 6C and 7C).^58^ Confocal imaging of hippocampus and cerebellar cortex (dotted boxed areas) confirmed high-resolution correlation between Nissl/DAPI morphology and GFAP reactivity (boxed regions) together with preservation of the brain anatomy, cytoarchitecture, and antigen immunoreactivity at a micrometer scale.

Fixation and washing steps before GFAP immunostaining successfully removed the matrix without compromising antigen binding and staining with blue-fluorescent DNA dye (Figures 6B.1–3 and 7B.1–4). Overall, data provided evidence for a high performance of the sucrose-based hydrogel matrix embedding procedure to preserve brain cell phenotypes and immunogenic reactivity such as those revealed for astrocytes and cell nuclei labeling by specific GFAP/DAPI binding.

## Conclusions

This study establishes a rigorously optimized sucrose–gelatin hydrogel embedding matrix as a robust and biocompatible platform for the desorption electrospray ionization-mass spectrometry imaging (DESI-MSI) of small, delicate, and morphologically sensitive tissues. The matrix provides exceptional structural preservation, maintaining cytoarchitectural fidelity and immunoreactivity at both macroscopic and cellular levels while concurrently enhancing the mechanical properties required for high-precision cryosectioning. Notably, the matrix generates minimal spectral background with sucrose-derived ions being the exclusive contributors to ionization signals, thereby enabling intrinsic lock-mass correction without the need for exogenous calibrants. This intrinsic referencing capability ensures superior mass accuracy across extended acquisition periods, eliminating the risk of ion suppression and preserving the native molecular environment. Data obtained comparing embedded and fresh-frozen tissues further corroborate this point, showing improved signal-to-noise ratios with the sucrose–gelatin embedding approach (Supporting Information, Figures S2–S5).

The integration of this embedding system with high-resolution DESI-MSI workflows (25 μm lateral pixel size) facilitated the spatially resolved identification of key lipid classes, including phosphatidylcholines (PCs), phosphatidylinositols (PIs), phosphatidylethanolamines (PEs), phosphatidylserines (PSs), and sulfatides (STs), across anatomically distinct brain regions, in agreement with well-established neuroanatomical lipid distributions. The platform also enabled the high-resolution mapping of poorly ionizable metabolites, such as cholesterol, without any form of chemical derivatization or surface modification, thereby simplifying sample preparation and maintaining molecular integrity.

Critically, the implementation of targeted DESI-MS/MS directly on tissue allowed the structural elucidation of lipid species within specific regions of interest (ROIs), effectively addressing isobaric and isomeric identification, a key limitation in high-throughput MSI workflows. The capacity to link MS/MS fragmentation patterns to distinct anatomical regions advances the level of molecular detail and confidence of molecular annotations *in situ*.

Current investigations are focused on improving the spatial resolution of DESI-MSI and using the matrix to study physiological and pathological models. Such advances will further enhance the specificity and depth of molecular imaging, opening transformative opportunities in spatial metabolomics, systems neuroscience, and digital histopathology. Although the present work has been focused on the mouse brain as a model tissue to validate our procedure, we have also successfully tested the sucrose–gelatin embedding medium on other fragile, small, and delicate tissues, whose results will be reported in forthcoming papers.

In conclusion, the proposed sucrose–gelatin embedding medium offers a reliable and reproducible platform for tissue preparation in DESI-MSI workflows, addressing key challenges in molecular imaging of fragile biological specimens while enabling downstream histological and immunochemical analyses. The approach is novel in its combination of a low chemical background, structural preservation, and internal lock-mass calibration via sucrose-derived ion adducts (Italian Patent n.102025000028453).^60^

## Supplementary Material



## Notes

Additional DESI-MSI figure of sucrose–gelatin matrix blocks; TIC comparison between fresh-frozen and embedded brain sections; combined spectra and extracted ion chromatograms (XICs) of representative lipid species from fresh-frozen and embedded brain sections; mirror plot of cholesterol MS/MS spectrum compared with GNPS library (PDF)
