# Supplementary material for: Innovative Application of a Multifunctional Sucrose–Gelatin Hydrogel Matrix in Desorption Electrospray Ionization-Mass Spectrometry Imaging
Source: Anal Chem. 2025 Nov 7;97(45):25087–98. doi: 10.1021/acs.analchem.5c04063 (PMC12631731; doi:10.1021/acs.analchem.5c04063)
Supplement: Supplementary file 1 [file ac5c04063_si_001.pdf]

Supporting Information

Innovative Application of a Multifunctional Sucrose-Gelatin Hydrogel Matrix in DESI-Mass Spectrometry Imaging

Marcello Ziaco[a,], Giovanni Andrea Vitale[b], Giusi Barra[a], Brenda Marfella[a], Mario dell'Isola[a], Federica Albiani[a], Angela Grazioso[a], Giuliana Giamundo[a], Genoveffa Nuzzo[a], Emiliano Manzo[a], Carmela Gallo[a], Daniela Castiglia[a], Lucia Verrillo[c], Maria G. Miano[c], Luigia Cristino[a], Ivan Conte[b], Giuliana d'Ippolito[a,*] and Angelo Fontana[a,b]*

[a] Institute of Biomolecular Chemistry ICB, National Research Council CNR, Via Campi Flegrei 34, 80078 Pozzuoli, Italy

[b] Department of Biology, University of Napoli "Federico II", Via Cupa Nuova Cinthia 21, 80126 Napoli, Italy

[c] Institute of Genetics and Biophysics IGB, National Research Council of Italy CNR, Via Pietro Castellino 111, 80131 Napoli, Italy

*Corresponding authors: marcelloziaco@cnr.it and giuliana.dippolito@cnr.it

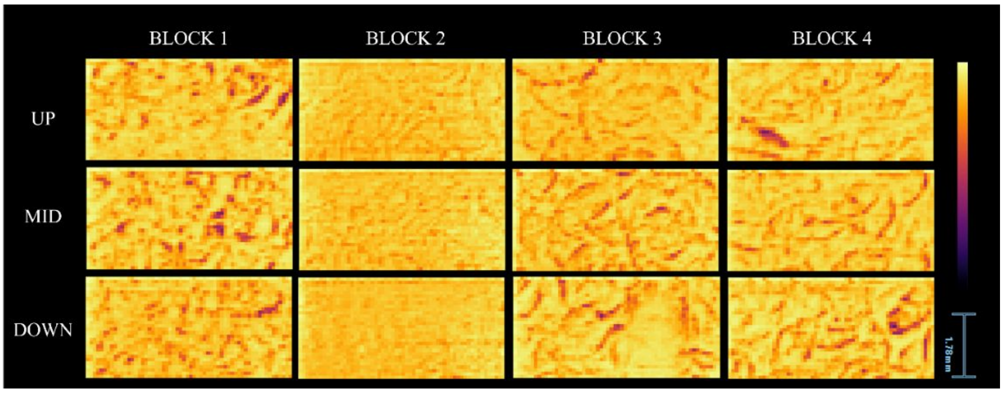

Figure S1. DESI-MSI analysis of four different matrix blocks, acquired in Full-MS negative ion mode, showing localization of deprotonated chlorinated adduct ion of sucrose ($[M+Cl]^-$, m/z 377.0856) performed on several sections at different block depths with thickness of 20 µm.

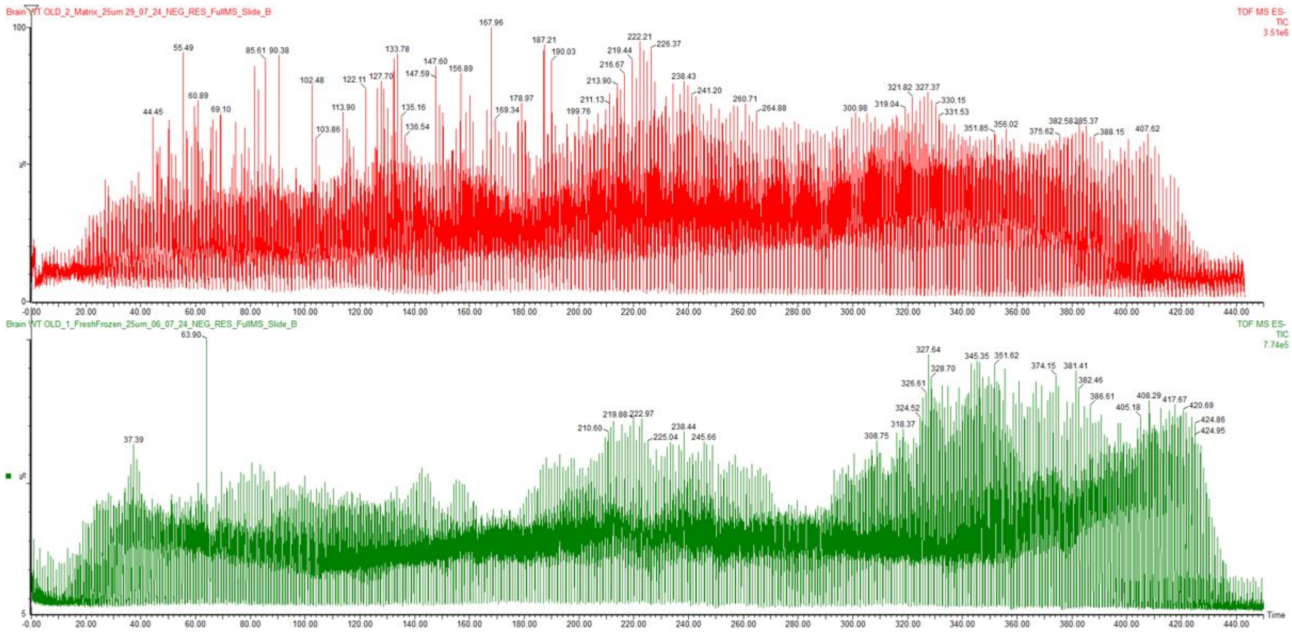

Figure S2. TIC comparison between fresh-frozen (green, bottom) and sucrose–gelatin embedded (red, top) mouse coronal brain sections, acquired in DESI full-MS negative ion mode at 25 µm lateral pixel size. The fresh-frozen section was analyzed with constant infusion of leucine enkephalin as lock-mass standard, whereas the embedded section was acquired without the use of leucine enkephalin. To ensure comparability, two coronal sections cut at similar distances from bregma and representing the same anatomical region were selected for analysis. For both samples, the acquisition pattern covered the entire tissue section using a rectangular pattern as scan area encompassing the whole brain. The spectra were obtained using the same experimental conditions (e.g solvent, solvent flow rate, sprayer adjustment, sprayer–tissue distance, and gas flow rate)

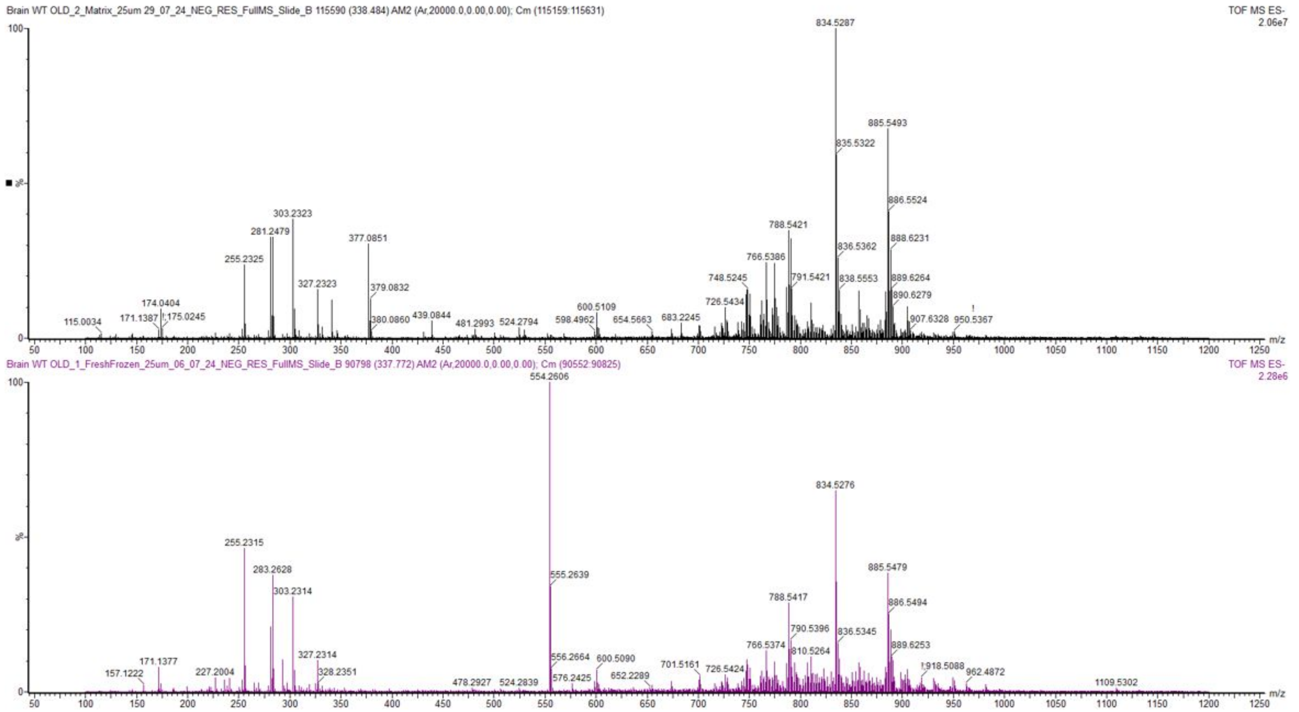

Figure S3. Comparison between fresh-frozen (purple, bottom) and sucrose–gelatin embedded (black, top) wild-type mouse coronal brain sections. The spectra were generated by combining all pixels from a single acquisition row in each section, selected at the same retention time and representing anatomically comparable regions of the brain. Both datasets were acquired under identical instrumental conditions in DESI full-MS negative ion mode at 25 µm lateral pixel size. The fresh-frozen section was analyzed with constant infusion of leucine enkephalin as lock-mass standard, whereas the embedded section was acquired without the use of leucine enkephalin.

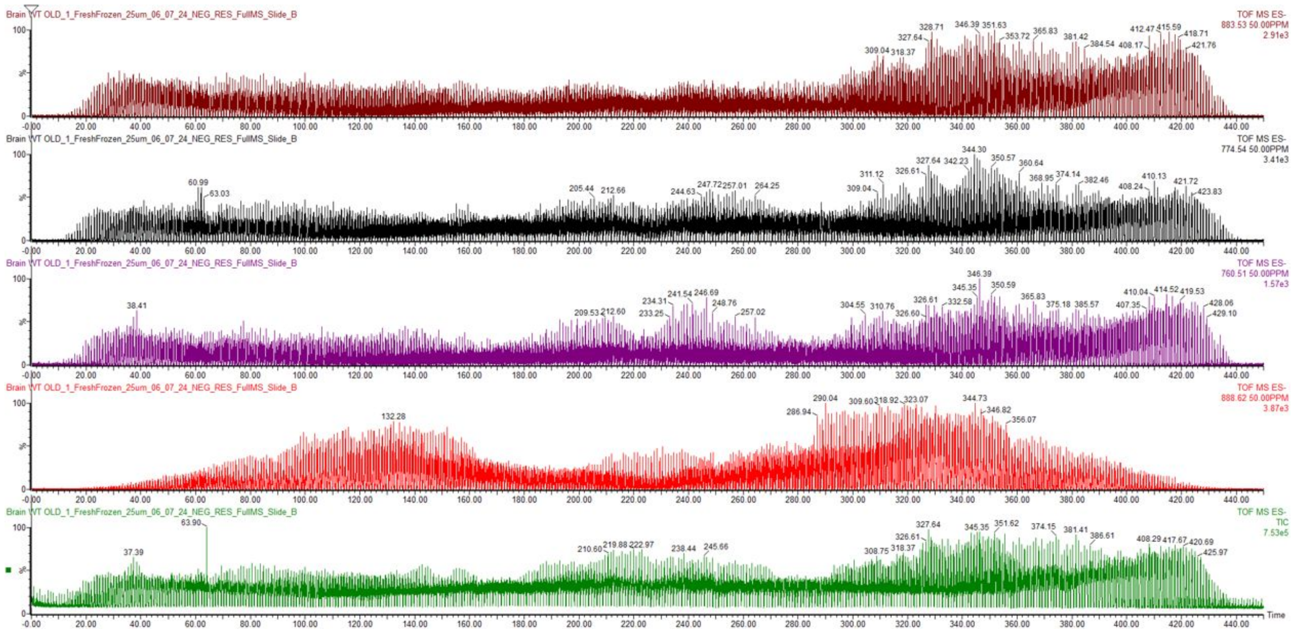

Figure S4. Extracted ion chromatograms (XICs) of representative lipid species obtained from the TIC (green) of a fresh-frozen mouse coronal brain section acquired in DESI full-MS negative ion mode at 25 µm lateral pixel size (mass range 100–1200 Da). The plots show XICs corresponding to some major lipids: phosphatidylinositol (PI 18:1/20:4, *m/z* 883.53, maroon), phosphatidylethanolamine (PE 18:0/22:6, *m/z* 774.54, black), phosphatidylserine (PS 16:0/18:1, *m/z* 760.51, purple), and sulfatide (ST d18:1/24:1, *m/z* 888.62, red).

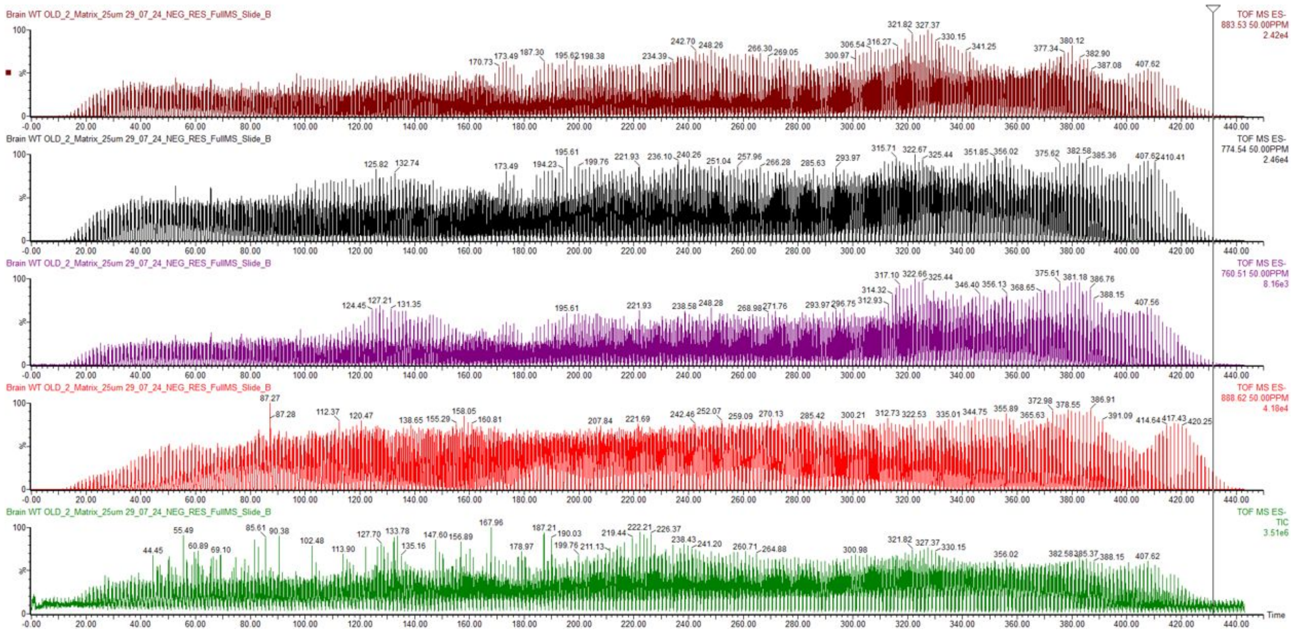

Figure S5. Extracted ion chromatograms (XICs) of representative lipid species obtained from the TIC (green), of a mouse coronal brain section embedded in sucrose–gelatin acquired in DESI full-MS negative mode at 25 µm lateral pixel size (mass range 100–1200 Da). The plots show XICs corresponding to four major lipid classes: phosphatidylinositol (PI 18:1/20:4, *m/z* 883.53, maroon), phosphatidylethanolamine (PE 18:0/22:6, *m/z* 774.54, black), phosphatidylserine (PS 16:0/18:1, *m/z* 760.51, purple), and sulfatide (ST d18:1/24:1, *m/z* 888.62, red).

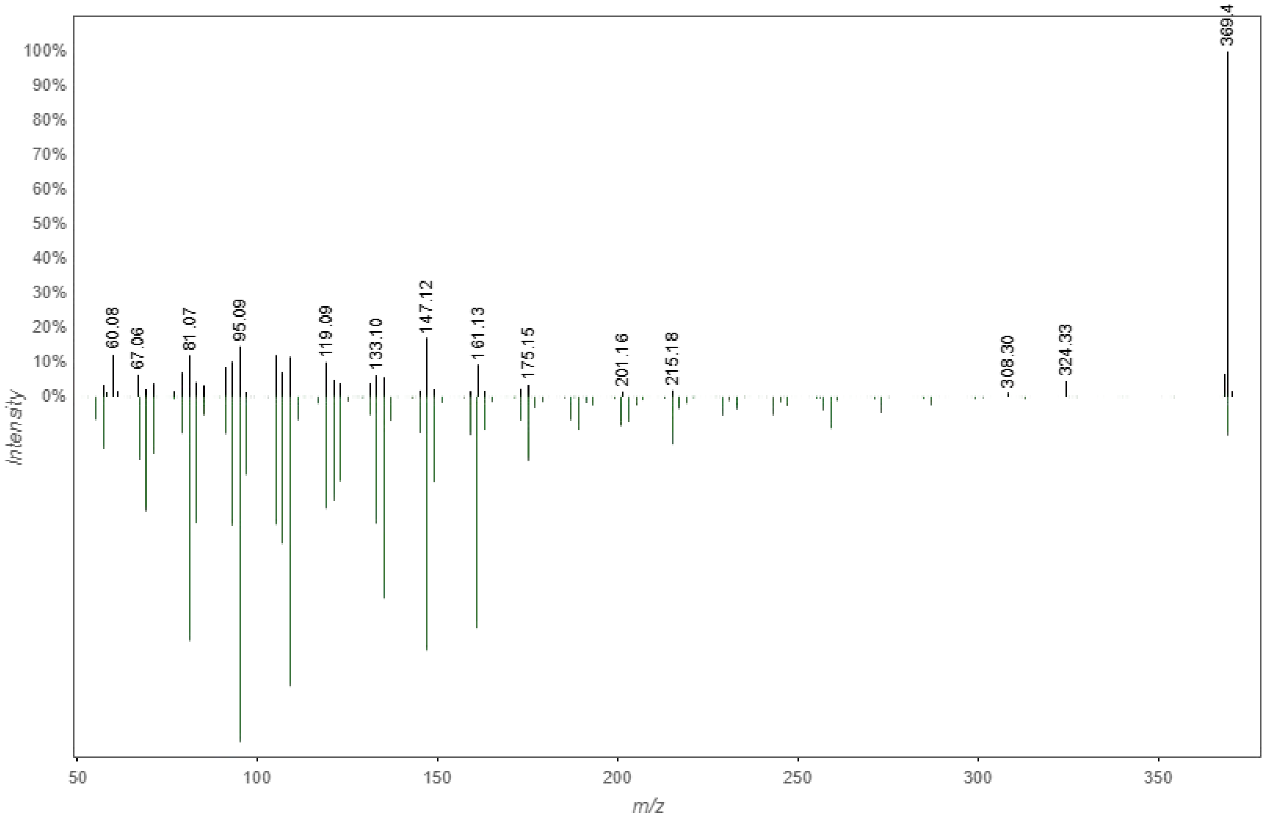

Figure S6. Mirror plot of cholesterol MS/MS spectra ($[M-H_2O+H]^+$; m/z 369.3516). The experimental spectrum (black, top) was acquired by DESI-MS/MS on a sucrose–gelatin embedded mouse brain section, targeting the dehydrated protonated ion of cholesterol. The reference spectrum (green, bottom) is the ESI-MS/MS library entry from GNPS. The close correspondence between the two (level-2 annotation; 33 shared peaks in the mirror plot) confirms the assignment of cholesterol in the embedded tissue.
